# Positionspapier Schlaganfallnachsorge der Deutschen Schlaganfall-Gesellschaft – Teil 2: Konzept für eine umfassende Schlaganfallnachsorge

**DOI:** 10.1007/s00115-021-01232-8

**Published:** 2021-12-21

**Authors:** Benjamin Hotter, Benno Ikenberg, Stephen Kaendler, Petra Knispel, Martin Ritter, Dirk Sander, Christopher Schwarzbach, Hans Joachim von Büdingen, Markus Wagner, Andreas Meisel, Mitglieder Kommission Nachsorge der Deutschen Schlaganfall-Gesellschaft, Heinrich Audebert, Hans Joachim von Büdingen, Joseph Claßen, Andreas Dreßing, Matthias Elstner, Armin Grau, Benjamin Hotter, Benno Ikenberg, Stephen Kaendler, Petra Knispel, Andreas Meisel, Dominik Michalski, Dirk Sander, Christopher Schwarzbach, Markus Wagner, Tobias Winkler

**Affiliations:** 1 Centrum für Schlaganfallforschung Berlin und Klinik und Hochschulambulanz für Neurologie, Charité – Universitätsmedizin Berlin, corporate member of Freie Universität Berlin, Humboldt-Universität zu Berlin, and Berlin Institute of Health (BIH), Charitéplatz 1, 10117 Berlin, Deutschland; 2 Neurologische Klinik und Poliklinik, Klinikum rechts der Isar, Technische Universität München, München, Deutschland; 3 Praxis Kaendler & Wurtz, Offenbach, Deutschland; 4 Servicepunkt Schlaganfall, Berliner Schlaganfall-Allianz e. V., Berlin, Deutschland; 5 Klinik für Schlaganfall- und Beatmungsmedizin, Clemenshospital, Münster, Deutschland; 6 Praxis Böckenholt & Ritter, Münster, Deutschland; 7 Neurozentrum Tutzing-Feldafing, Benedictus-Krankenhaus, Tutzing, Deutschland; 8 Klinik für Neurologie, Klinikum Ludwigshafen, Ludwigshafen, Deutschland; 9 Neurozentrum Ravensburg, Ravensburg, Deutschland; 10 Stiftung Deutsche Schlaganfall-Hilfe, Gütersloh, Deutschland

**Keywords:** Schlaganfall, Nachsorge, Versorgungsforschung, Comprehensive Care, Sekundärprävention, Long-term care, Stroke, Delivery of health care, Comprehensive health care, Secondary prevention

## Abstract

Die Schlaganfallnachsorge ist im Gegensatz zur akuten und rehabilitativen Versorgung des Schlaganfalls wenig standardisiert. Der fragmentierte ambulante Sektor erlaubt hierbei ein hohes Maß an Flexibilität, leidet aber folglich an variabler Qualität der Nachsorge. Die Kommission Nachsorge der Deutschen Schlaganfall-Gesellschaft formuliert in diesem Positionspapier ein inhaltliches Konzept, um eine strukturierte Nachsorge mit multiprofessionellem Ansatz zu entwickeln. Diese soll im Sinne einer „Comprehensive-care“-Versorgung und patientenzentriert erfolgen. Dazu schlagen wir ein diagnostisches Stufenkonzept mit Screening und ggf. weitergehender Untersuchung vor, das in Absprache mit den Betroffenen zu einem standardisierten Therapieplan führt, der im Langzeitverlauf entsprechend angepasst werden muss. Inhaltlich sind sowohl internistische Domänen (Management von Risikofaktoren) als auch genuin neurologische Domänen (Spastik, kognitive Defizite etc.) zu berücksichtigen. Besondere Herausforderungen an dieses Konzept sind die sektorenübergreifende (inter- und intrasektorale) Kommunikation zwischen den Akteuren im Gesundheitswesen untereinander sowie mit den Patienten und Angehörigen, die Notwendigkeit zur Schaffung eines Vergütungsmodells für eine solche Nachsorge und letztlich die Etablierung eines entsprechenden Qualitätsmanagements. Digitale Lösungen erachten wir als hilfreiche Werkzeuge für Aspekte der Diagnose, Therapie und Kommunikation in der Schlaganfallnachsorge.

## Abstract (en)

Long-term management after stroke has not been standardized in contrast to acute and rehabilitative stroke care. The fragmented outpatient sector of healthcare allows a high degree of flexibility but also variability in the quality of care provided. The commission on long-term stroke care of the German Stroke Society developed a concept on how to provide standardized multiprofessional long-term stroke care with a focus on patient-centered comprehensive care. We propose a diagnostic work-up in stages, with an initial patient-reported screening, followed by an adapted in-depth assessment of affected domains. This includes internistic domains (management of risk factors) and also genuine neurological domains (spasticity, cognitive deficits etc.) that must be considered. This information is then merged with patient expectations and prioritization to a standardized treatment plan. Special challenges for the implementation of such a comprehensive care system are the intersectoral and intrasectoral communication between healthcare providers, patients and relatives, the need to create a compensation system for providers and the establishment of appropriate quality management services. Digital health applications are helpful tools to provide aspects of diagnostics, treatment and communication in long-term stroke care.

In Deutschland erleiden jährlich etwa 270.000 Menschen einen Schlaganfall, wovon 70.000 ein Rezidivereignis erleiden. In Deutschland beantragt ein Fünftel der Schlaganfallpatienten Leistungen für die postakute Behandlung bei ihren Krankenkassen. Auf Leistungen der Frührehabilitation und Anschlussheilbehandlung entfallen etwa 37 % dieser Kosten, sodass der Großteil des ökonomischen Aufwands in den Folgejahren bei der ambulanten Versorgung entstehen [7]. Im fragmentierten ambulanten Sektor fehlt allerdings ein „Comprehensive-care“-Modell für eine nachhaltige, patientenzentrierte Nachsorge [10].

## Stand der Nachsorge und Struktur

Für die Phasen der Akutversorgung und frühen Rehabilitation existieren Maßnahmen zur Standardisierung und zum Qualitätsmanagement. Die Nachsorge wurde wissenschaftlich wenig untersucht. Es fehlen interdisziplinäre Ansätze in Deutschland, wobei in Pilotprojekten modellhafte Lösungen entwickelt werden (Tab. 1). Die Europäische Schlaganfallorganisation priorisiert seit 2018 die Entwicklung von Nachsorgekonzepten unter der Domäne „Life after Stroke“ im Stroke Action Plan [15]. In Kanada wurden bereits konkrete Empfehlungen definiert [26]. In Deutschland hat die Leitlinie der Deutschen Gesellschaft für Allgemeinmedizin und Familienmedizin Empfehlungen für die Schlaganfallnachsorge entwickelt [5].

**Table 1:** 

**National**		
Projekte	INSPiRE-TMS [1]	Sekundärpräventionsambulanz
	INVADE (www.invade.de)	
	MAS [10]	Ambulanz mit „Comprehensive-care“-Ansatz
	SANO (www.sano-studie.de)	Strukturiertes, sektoren- und berufsgruppenübergreifendes Nachsorgeprogramm
	SPS-BSA [16]	Sozialarbeiterische Beratungsstelle zur Schlaganfallnachsorge
	Stroke Nurse [22]	Sektorenübergreifende Schlaganfallnachsorge durch spezialisierte Krankenschwester
	SOS-Care [3]	(Digitale) Schlaganfall-Lotsen/Case-Management-Programme
	STROKE OWL (www.stroke-owl.de)	
	HANNS (www.klinikum-hanau.de)	
	Poststroke-Manager (www.iccas.de/poststroke)	
Leitlinien	DEGAM-Leitlinie Schlaganfall [5]	Ausführliche Empfehlungen zur rehabilitativen und Anschlussversorgung für Allgemeinmediziner
**International**		
	ESO/SAFE Stroke Action Plan-Europe [15]	Nachsorge mit „Life after Stroke“ als eine von 7 zentralen Domänen identifiziert
	Canadian Stroke Best Practices [26]	Module „Secondary Prevention“, „Community Participation“ und „Activity“, Versorgungssäule „Stroke Management in Long-Term-Care“

**Figure 1:**
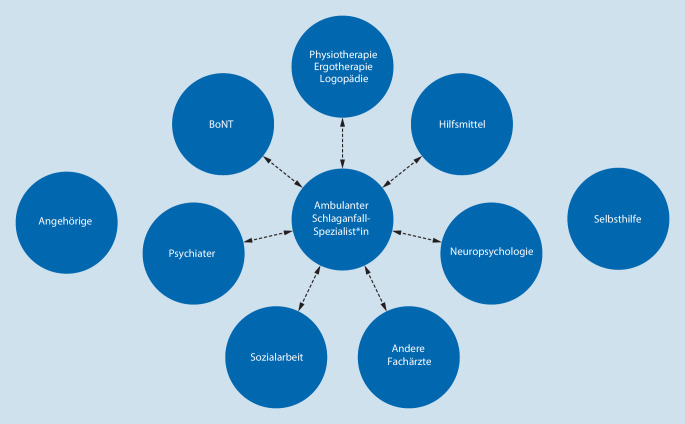

In der Versorgung der Schlaganfallpatienten, insbesondere in der Nachsorge, spielen neben den professionellen Akteuren des Gesundheitswesens die Angehörigen sowie Selbsthilfegruppen eine zentrale Rolle (Abb. 1). Die Nutzung der unterschiedlichen Versorgungs- und Unterstützungsangebote des Gesundheits- und Sozialsystems setzt jedoch ein hohes Maß an Koordinationsleistungen voraus, mit denen Patienten und Angehörige regelhaft überfordert sind. Von zentraler Bedeutung für die Schlaganfallnachsorge ist ein Spezialist, der die spezialisierte Behandlung bedarfsgerecht in interdisziplinärer Zusammenarbeit koordiniert (Abb. 1). In der aktuellen Versorgungswirklichkeit soll diese sog. Lotsenfunktion der Hausarzt übernehmen, die dieser jedoch aufgrund fehlender Voraussetzungen nicht erfüllen kann [2]. Zum aktuellen Stand der Schlaganfallnachsorge in Deutschland und deren Problemen sowie zu Vorschlägen für strukturelle Verbesserungen verweisen wir auf die zwei begleitenden Arbeiten der Kommission Nachsorge der Deutschen Schlaganfall-Gesellschaft in dieser Ausgabe. Wir wollen uns hier im Folgenden auf die inhaltlichen Aspekte der Schlaganfallnachsorge fokussieren. Diese liegen überwiegend im neurologischen Fachgebiet, das damit eine Schlüsselposition nicht nur in der Entwicklung, sondern auch Umsetzung des Nachsorgekonzepts einnimmt. Die notwendige Expertise ist durch das Facharztkurrikulum neurologisch verortet. Aus logistischen und gesundheitsökonomischen Gründen kann diese jedoch auch über eine zu schaffende Zusatzqualifikation hausärztlich oder anderenorts abgebildet werden. Voraussetzung für die praktische Umsetzung ist eine qualitätsbasierte Vergütung der Nachsorge wie dies auch die Voraussetzung für die erfolgreiche Implementierung des Stroke-Unit-Konzepts in der Akuttherapie war.

## Systemische Anforderungen

Der Erfolg der Schlaganfallnachsorge hängt von der Vernetzung und dem kommunikativen Austausch zwischen den beteiligten professionellen Akteuren untereinander und den Betroffenen (Patienten, Angehörigen) ab, um auf (veränderte) Untersuchungsbefunde bedarfsgerecht zu reagieren, die Patienten zu bestärken („empowerment“) und die Adhärenz zu erhöhen. In Anbetracht des hochgradig fragmentierten ambulanten Gesundheitssektors ist dies eine besondere Herausforderung für alle Beteiligten in der Schlaganfallnachsorge. Um die konsequente Umsetzung der Schlaganfallnachsorge qualitätsbasiert zu ermöglichen, bedarf es eines aufwandgerechten Vergütungsmodells für die Leistungserbringer entlang der gesamten Versorgungskette. Hierfür sieht die Kommission die Notwendigkeit, einen ergänzenden Leistungskatalog für die Vergütung der umfassenden Versorgung zu definieren und einzuführen.

Für die erfolgreiche Umsetzung und Entwicklung der Schlaganfallnachsorge müssen Maßnahmen der Qualitätssicherung flankierend implementiert werden. Dazu müssen frühzeitig praktikable Qualitätsindikatoren unter wissenschaftlicher Begleitung entwickelt werden. Analog zur Akutphase schlägt die Kommission die Etablierung eines Nachsorgeregisters auf Basis ausgewählter Indikatoren unter Nutzung von Routine- sowie Outcomedaten für die Qualitätssicherung vor, das sich an der Konzeptskizze des aQua-Instituts für den Gemeinsamen Bundesausschuss orientiert. Auf Basis solcher Daten kann auch eine Zertifizierung der Versorger in unterschiedlichen Qualifikations- bzw. Leistungsstufen erfolgen, die die primäre Nachsorge (z. B. Praxen), diagnostische Zentren (z. B. spezialisierte Ambulanzen) und tertiäre Zentren (z. B. Spezialdiagnostik oder -therapie) umfassen.

## Konzeptionelle Struktur der Visite

Ziel der Erstvorstellung (ggf. nach Rehabilitation) bei den niedergelassenen Schlaganfallspezialisten ist die

- Klärung der adäquaten Sekundärprophylaxe und deren korrekte Umsetzung,
- umfassende Abklärung versorgungsrelevanter Defizite und Komplikationen des Schlaganfalls,
- Indikationsstellung für ggf. zusätzliche (apparative) Diagnostik bei unklaren Befunden,
- Vereinbarung von Therapiezielen mit Patienten und Angehörigen,
- konkrete Erstellung eines Therapieplans inkl. Heil- und Hilfsmittelversorgung mit entsprechender Rezeptierung,
- Terminierung der Folgeuntersuchung.

**Figure 2:**
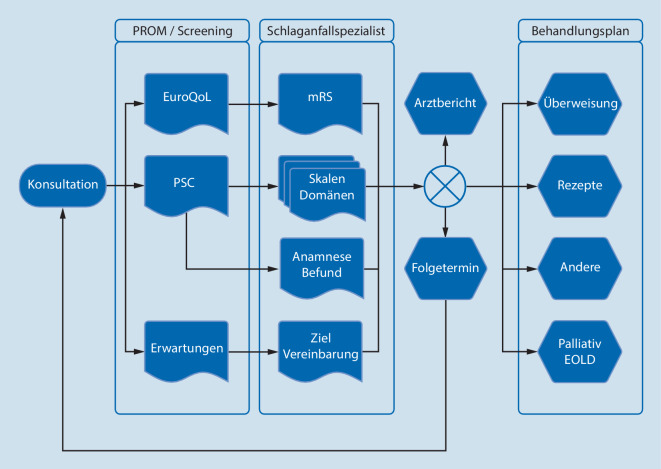

Hinsichtlich der Sekundärprophylaxe wird auf die entsprechenden Leitlinien verwiesen, deren Aktualisierung in der ersten Jahreshälfte 2021 erwarten wird [8]. Um die Umsetzung der Empfehlungen zu gewährleisten, sollte an der Schnittstelle zwischen stationärem und ambulantem Sektor eine zu entwickelnde standardisierte Checkliste genutzt werden. Im Rahmen der Verlaufsvorstellungen sind bedarfsgerecht weitere Untersuchungen notwendig. Um der Komplexität der multiplen Domänen mit möglichem Bedarf gerecht zu werden und gleichzeitig ökonomisch realistischen Aufwand zu berücksichtigen, ist ein mehrstufiges Vorgehen (Screening mit ggf. Folgediagnostik im Sinne eines Stufenschemas) notwendig (Abb. 2).

### Stufendiagnostik

Hierbei können der Patient und seine Angehörigen selbstständig (z. B. im Wartezimmer) die Fragebögen zur Lebensqualität (EuroQoL) und ein Screeningdokument für spezifische Defizite (Post-Stroke-Checklist, PSC) ausfüllen (siehe auch Tab. 2). Der EuroQoL dient zur Erfolgskontrolle der Nachsorge, ist hinsichtlich der Erhebung und Vergleichbarkeit von den individuell betroffenen Domänen unabhängig und bietet sich als Qualitätsindikator im Sinne eines Outcomeparameters an. Die PSC [17] ergibt mit einfachen Fragen einen raschen Überblick über etwaige Defizite und ist aufgrund der besseren Validierung dem Greater Manchester-Stroke Assessment Tool [20] vorzuziehen. Die geringe Komplexität der PSC erhöht die Anwendbarkeit auch im hausärztlichen Bereich. Darüber hinaus sollte ein Screening für die Gebrechlichkeit mittels Clinical Frailty Scale [19] vorgenommen werden, über die auch Domänen wie Stürze, Inkontinenz und Sensorikstörungen miterfasst werden können.

**Table 2:** 

Domäne	Assessment	Cut-Offs	Im Screening erfasst
Lebensqualität	*EuroQoL*	–	–
Behinderung	Modified Rankin Scale Barthel-Index	–	–
Screening	*Post-Stroke-Checkliste*	–	(✓)
Spastik	Modified Ashworth Scale	mAS ≥3 in ≥1 Gelenk (nicht validiert)	✓
Aphasie	Token Test	>3	✓
Kognitive Defizite	Montreal Cognitive Assessment	<26	✓
Depression	Hamilton Depression Scale	>8	✓
	*Beck-Depressionsinventar II *[11]	≥9	
Sozialer Bedarf	Nikolaus Soziale Situation	<17	✓
Familiäre Belastung	Häusliche Pflegeskala – kurz	>9	✓
Schmerz	Visuelle Analogskala	1–10	✓
	*Ggf. PainDetect*	>19 sicher 13–18 unklar 0–12 negativ	
Sexualfunktion	*Quality of Sexual Function Scale *[9]	–	–
Fatigue	*Fatigue Assessment Scale *[13]	≥22	–
Sensorik (Sehen/Hören)	–	–	–
Dysphagie	Bogenhausener Dysphagiescore [4]	≥3	–
	Fiberoptischer endoskopischer Dysphagieschweregradscore [24]	≥2	
Malnutrition	–	–	–
Sturzprävention	–	–	–
Gebrechlichkeit	Clinical Frailty Scale	≥5	–
Fahrtüchtigkeit	Positionspapier DGNR/DGN/DGNC/DSG/GNP [12]	–	–

*Kursiv* dargestellt sind Instrumente die als PROM z. B. bereits im Wartezimmer durch die Patienten vorbereitet werden können. Sofern keine Quelle angegeben, siehe [10]

*mAS* modified Ashworth Scale, *DGNR* Deutsche Gesellschaft für Neurorehabilitation, *DGN* Deutsche Gesellschaft für Neurologie, *DGNC* Deutsche Gesellschaft für Neurochirurgie, *DSG* Deutsche Schlaganfall-Gesellschaft, *GNP* Gesellschaft für Neuropsychologie

### Neurologische Domänen

Die Untersuchung durch die Spezialisten baut auf den Screeningschritten auf. Neben der Anamnese und klinisch-neurologischen Untersuchung erfolgt eine tiefergehende Diagnostik in den Domänen mit Hinweisen für Defizite. Sollten diese spezifischen Instrumente pathologische Befunde ergeben, leiten sich daraus Behandlungsindikation ab (Tab. 2). Details zu den empfohlenen Instrumenten und Grenzwerte zur Stellung der Therapieindikation werden im nächsten Abschnitt beschrieben.

### Sekundärprophylaxe

Zunächst muss geprüft werden, ob alle notwendigen Befunde zur ätiologischen Zuordnung des Schlaganfalls in aktueller Form vorliegen. Die Zuordnung des Ereignisses zu einer klar definierten Kategorie (z. B. TOAST-Kriterien o. Ä.) muss angestrebt werden, da diese wesentliche Implikationen für das Rezidivrisiko und die entscheidenden Akzente in der Sekundärprävention hat. Hierzu zählen die Duplexsonographie, (Langzeit‑)Blutdruckmessung, 12-Kanal- und ggf. Langzeit-EKG, Echokardiographie sowie eine labordiagnostische Risikostratifizierung (Lipoproteine, Glukosestoffwechsel). Seltene Ursachen (Dissektionen, Koagulopathien, genetische Ursachen) und relevante Differenzialdiagnosen müssen ggf. erneut berücksichtigt werden. Für die zu erhebenden Parameter sollte ebenfalls eine einheitliche Checkliste entwickelt werden. Hierbei sollten auch sog. Lifestylefaktoren, wie schädlicher Gebrauch von Genussmitteln (Nikotin, Alkohol etc.) sowie körperliche Betätigung und Ernährung berücksichtigt werden.

### Zielvereinbarung und Kontrolle

Im Anschluss sollte ein Gespräch über die Erwartungshaltung und den Wissensstand der Patienten erfolgen. Patienten und Angehörige teilen nicht zwangsläufig die Behandlungsprioritäten der Ärzte. Im Rahmen der erreichbaren Ziele sollte die Priorisierung der Betroffenen im Vordergrund stehen, um der Komplexität der individuellen Situation Rechnung zu tragen. Darüber hinaus sollten explizit palliative Konzepte, Patientenverfügungen und Vorsorgevollmachten angesprochen werden.

Die folgenden Nachsorgetermine hängen von den diagnostischen und therapeutischen Bedarfen ab, die Nachsorge sollte jedoch wenigstens in halbjährlichen Abständen erfolgen.

## Instrumente

Nach Screening auf bestehende Defizite sollten auffällige Befunde mit spezifischen Instrumenten verifiziert und quantifiziert werden. Bei pathologischen Befunden ergibt sich eine relative Behandlungsindikation, wobei der subjektive Leidensdruck der Patienten auf Basis der jeweiligen Defizite unter Berücksichtigung erreichbarer Therapieziele relevanter Schlüssel zur festen Formulierung der Indikation wird. Einen Überblick gibt Tab. 2.

### Lebensqualität und Grad der Behinderung – EuroQoL, mRS, BI

Diese Skalen geben einen domänenunabhängigen Überblick über die Beeinträchtigung der Patienten, erlauben die Quantifizierung von Therapieeffekten und sind in weiterer Folge zentrale Ansatzpunkte für die Entwicklung von Qualitätsindikatoren.

### Individuelle Domänen

1. *Spastik – modifizierte Ashworth Scale (mAS)*
  Die Literatur diskutiert ideale Skalen zur Spastikerhebung kontrovers (insbesondere hinsichtlich der Schnittstelle zu Schmerz, Immobilität und Funktionsverlust). Aus pragmatischen Gründen empfehlen wir die modifizierte Ashworth-Skala zur Indikationsstellung einer fokalen oder systemischen antispastischen Therapie.
2. *Post Stroke Schmerz – PainDetect*
  Die PainDetect-Skala ist ein schnelles Screeningtool für neuropathischen Schmerz mit validierten Grenzwerten. Kritisch wird angemerkt, dass die PainDetect-Skala für peripher-neuropathische Schmerzen entwickelt wurde und möglicherweise keinen relevanten zusätzlichen Nutzen zur anamnestischen Angabe von Schmerzen über die Wahl des Präparates hinaus hat.
3. *Aphasie – partieller Aachener Aphasietest: Token Test und Schriftsprache*
  Der Token Test ist ein schneller Screeningtest für Aphasie, erfasst jedoch keine Defizite der Schriftsprache. Hierfür kann die Diagnostik um die übrigen Teile des Aachener Aphasietests ergänzt werden. Beide Tests benötigen Training, sodass bei klinischem Verdacht die formelle Testung an Spezialisten übertragen werden sollte.
4. *Kognitive Defizite – Montreal Cognitive Assessment (MoCA) bzw. CERAD (Consortium to Establish a Registry for Alzheimer’s Disease)*
  Der MoCA-Test ist für *„mild cognitive impairment“* sensitiver als die bekanntere Mini Mental State Examination oder der in Praxen weit verbreitete DEMTECT. Alternativ kann der ausführlichere CERAD-Test eingesetzt werden, der eine Differenzierung direkt betroffener Teildomänen erlaubt.
5. *Depression – Hamilton Depression Scale (HAMD-17) bzw. Beck-Depressionsinventar (BDI-II)*
  Bei positivem Screening sollte für die Diagnosestellung die Hamilton-Depressionsskala erhoben werden. Zusätzlich kann das Beck-Depressionsinventar für die Beurteilung der Schwere einer Depression genutzt werden (BDI-II), das als PROM durch den Patienten selbst ausgefüllt wird.
6. *Sozialer Bedarf – Soziale Situation nach Nikolaus (SoS)*
  Der Fragebogen nach Nikolaus ist stellenweise veraltet, erlaubt jedoch mittels einer klinisch validierten Skala die zügige Entscheidung, ob sozialarbeiterischer Beratungsbedarf besteht.
7. *Familiäre Belastung – Burden Scale for Family Caregivers (BSFC)*
  Die häusliche Pflegeskala ist ein kurzer Fragebogen, den die engsten Angehörigen der Patienten ausfüllen. Hier wird schnell ersichtlich, ob zusätzlicher Bedarf an pflegerischer Unterstützung bzw. Umbauten in der Häuslichkeit der Patienten notwendig sind.
8. *Sexualfunktion – Quality of Sexual Function Scale (QOFS)*
  Etwa 75 % aller Schlaganfallpatienten berichten von Störungen der sexuellen Funktion. Häufig wird die Thematik jedoch aus Scham nicht angesprochen. Die QOFS bietet als PROM die Möglichkeit, niederschwellig etwaige Bedürfnisse zu diagnostizieren.
9. *Fatigue – Fatigue Assessment Scale (FAS)*
  Fatigue ist eine wesentliche Begleiterscheinung vieler chronischer Erkrankungen und beeinträchtigt weite Teile der täglichen Aktivitäten der Patienten. Für Schlaganfallpatienten wurde die FAS im direkten Vergleich validiert.
10. *Dysphagie – Bogenhausener Dysphagiescore (BODS) bzw. fiberoptischer endoskopischer Dysphagieschweregradscore (FEDSS)*
  Mittels BODS ist die Schwere einer Dysphagie zu bewerten, aber keine Handlungsempfehlung abzuleiten. Der FEDSS ist durch den Bedarf einer fiberendoskopischen Untersuchung aufwendiger, ergibt jedoch eine klare Kostform bzw. Indikation zur nasogastralen Sondierung. Beide Instrumente benötigen eine entsprechende Weiterbildung. Damit fehlt für die Praxis neben der klinischen Untersuchung ein einfaches, validiertes Instrument zum sicheren Ausschluss einer Dysphagie.
11. *Sensorik, Malnutrition und Sturzprävention*
  Es fehlen validierte Skalen für diese Domänen, sodass diese Domänen exklusiv durch die klinische Untersuchung abgedeckt werden müssen.

## Therapien – Interventionen

Aus den erhobenen Befunden, Defiziten und Komplikationen leitet sich die standardisierte Therapieempfehlung inklusive Sekundärprophylaxe ab (Tab. 3). Dabei werden bestimmte Aufgaben gezielt an andere Akteure des Gesundheitswesens übergeben (Therapeuten, andere Fachärzte) und Leistungen im (regionalen) Versorgungsnetzwerk koordiniert.

**Table 3:** 

Domäne	Therapie	Leitlinie	Mögliche Akteure
Motorisches Defizit	Physio‑/Ergotherapie Ggf. spezifische Therapien (z. B. Neurourologie)	[14]	Therapeuten Fachärzte
Signifikante Spastik	Physiotherapie Botulinumneurotoxin, orale Relaxanzien	[18]	Therapeuten Neurologen
Post-Stroke-Schmerz	Multimodale analgetische Therapie Überweisung zu schmerztherapeutischem Zentrum	[21]	Neurologen Schmerzzentren
Aphasie	Sprachtherapie	([25], abgelaufen)	Neuropsychologen Logopäden
Kognitive Defizite	Kognitives Training Ggf. Psychotherapie (affektive Modulation)	[23]	Neuropsychologen Psychotherapeuten
Depression	Antidepressiva Psychotherapie	[6]	Neurologen Psychiater Psychotherapeuten
Adhärenz	Verhaltensempfehlungen (Medikationsplatzierung, Angehörige) Technische Lösungen (Smartphone-Erinnerungen, „smart pillbox“)	–	Hausärzte Pflegestützpunkt
Soziale Arbeit	Sozialrechtliche Ansprüche Psychosoziale Bedürfnisse	–	Sozialarbeiter Pflegestützpunkt
Angehörige	Unterstützung durch (häusliche) Pflege Selbsthilfegruppen Psychotherapie u./o. Paarberatung	–	Pflegestützpunkt Selbsthilfegruppen Psychotherapeuten
Medizinische Faktoren	Sekundärprophylaxe Überweisung Spezialsprechstunden (juveniler Stroke, V. a. seltene Ursachen)	[8]	Neurologen Hausärzte Fachärzte

## Kommunikation

Die Visitenberichte sollten in knapper tabellarischer Form gehalten sein, um weiterbehandelnden Versorgern einen schnellen Überblick zu ermöglichen. Eine einfache Checkliste (Beispiel in Tab. 4), die eine visuelle Orientierung über die vorliegenden Befunde und Therapieempfehlungen erlaubt, sollte für die zukünftige Versorgungsroutine genutzt werden.

**Table 4:** 

Domäne	Behandlung	Ja	Nein	Abgelehnt	Nicht betroffen
Sekundärprophylaxe	Medikationswechsel	□	□	□	□
	Verhaltensempfehlungen (Adhärenz)	□	□	□	□
	Devices („smart pillbox“)	□	□	□	□
	Täglicher Besuch (ggf. Pflegedienst)	□	□	□	□
Mobilität	Physiotherapie	□	□	□	□
	Ergotherapie	□	□	□	□
	Hilfsmittel/Umbauten	□	□	□	□
Spastik	Orale Muskelrelaxanzien	□	□	□	□
	Fokale Therapie (BoNT)	□	□	□	□
	Physiotherapie	□	□	□	□
Schmerz	NSAID	□	□	□	□
	Antikonvulsiva/Antidepressiva	□	□	□	□
	Lokale Analgetika	□	□	□	□
	Psychotherapie	□	□	□	□
	Schmerztherapeut	□	□	□	□
Kommunikation	Sprachtherapie/Logopädie	□	□	□	□
	Hilfsmittel	□	□	□	□
Stimmung	Antidepressiva	□	□	□	□
	Psychotherapie	□	□	□	□
Kognition	Ergotherapie	□	□	□	□
	Kognitives Training	□	□	□	□
	Vorstellung Neuropsychologie	□	□	□	□
Inkontinenz	Überweisung (Neuro‑)Urologie	□	□	□	□
Leben nach Stroke	Soziale Arbeit	□	□	□	□
	Hilfsmittel und Umbauten	□	□	□	□
Beziehungen	Soziale Arbeit	□	□	□	□
	(Paar‑)Therapie	□	□	□	□
Andere	Überweisung zu Spezialisten	□	□	□	□

## „Digital health“

Die Nutzung digitaler Lösungen für den effektiven Informationsfluss sollte in zukünftigen Versorgungsmodellen berücksichtigt werden. Hierdurch kann sowohl die (sektorenübergreifende) Kommunikation zwischen den Akteuren als auch mit den Patienten und Angehörigen verbessert werden. Letzteres kann beim Betroffenen die Resilienz und Einbindung in die Ausgestaltung ihrer Nachsorge („empowerment“) stärken. Digitale Lösungen können neben der Detektion von Risikofaktoren wie z. B. intermittierendem Vorhofflimmern durch externe mobile Geräte („wearables“) in der Praxis eine sinnvolle Ergänzung sein. Tele-Rehabilitation kann helfen, den Aufwand der standardisierten Erfassung von Symptomen, Risikofaktoren, Medikation und damit auch Qualitätsindikatoren (im Sinne sog. „electronic PROM“) zu reduzieren. Eine digitale Patientenedukation kann als Ergänzung zu Sekundärpräventionsprogrammen eingesetzt werden.

In den kommenden Jahren ist daher mit einer Reihe digitaler Gesundheitsanwendungen in der Nachsorge von Schlaganfällen und deren Risikofaktoren zu rechnen. Der Gestaltungsspielraum liegt hier nicht nur in der Entwicklung dieser Anwendungen, sondern vor allem auch in der sinnvollen Integration vorhandener Angebote in die praktische Versorgung, ggf. unter Nutzung von Dachplattformen. Voraussetzung ist die wissenschaftliche Evaluation und Entwicklung von Qualitätskriterien, die von der DSG koordiniert werden sollten.
